# Diagnostic accuracy of a modified non-contrast O-RADS MRI scoring system: a retrospective study

**DOI:** 10.1186/s13244-026-02425-y

**Published:** 2026-10-16

**Authors:** Yan Ren, Can Cui, Zhijing Zhang, Wei Liao, Chen Zhang, Yue Cheng, Wen Shen

**Affiliations:** 1 First Central Hospital of Tianjin Medical University, Tianjin, China; 2 Department of Radiology, Tianjin First Central Hospital, Tianjin, China; 3 Department of Radiology, Jinhua Municipal Central Hospital, Jinhua, China; 4 Department of Radiology, The No.988 Hospital of Joint Logistic Support Force of Chinese People’s Liberation Army, Zhengzhou, China; 5 School of Medicine, Nankai University, Tianjin, China; 6 MR Collaborations, Siemens Healthcare Ltd., Beijing, China; 7 Department of Radiology, First Central Hospital of Tianjin Medical University, Tianjin First Central Hospital, Tianjin Institute of Imaging Medicine, Tianjin, China

**Keywords:** Ovarian neoplasms, Risk assessment, Magnetic resonance imaging

## Abstract

**Objective:**

To develop a modified non-contrast O-RADS MRI scoring system and validate its diagnostic performance.

**Materials and methods:**

The modified non-contrast O-RADS MRI scoring system was developed by incorporating the significant signs from the standard O-RADS MRI system. This study retrospectively enrolled the 176 adnexal masses in cohort A. MRI imaging features were compared between benign and malignant tumors within this cohort. The diagnostic performance of the two scoring models was assessed by estimating the area under the curve (AUC), net reclassification improvement (NRI), and integrated discrimination improvement (IDI). In addition, 352 adnexal lesions from cohorts B and C were included to further evaluate the diagnostic ability of the modified scoring system.

**Results:**

In cohort A, the papillary projections exhibiting the sea anemone sign were highly indicative of borderline lesions (*p* = 0.001). Diffusion restriction in solid components significantly distinguished benign from malignant masses (*p* = 0.001). The apparent diffusion coefficient (ADC) value of cystic components yielded a cutoff of 1.74 (*p* < 0.05). The DeLong test revealed no statistically significant difference in AUCs between the two systems in cohort A (*p* > 0.05). The NRI and IDI for the modified non-contrast O-RADS MRI scoring system were −4.13% and −0.04, respectively (all *p* > 0.05). In cohorts B and C, the AUCs were 0.933 and 0.910.

**Conclusion:**

The diagnostic efficacy of the modified non-contrast O-RADS MRI scoring system was comparable to that of the standard O-RADS MRI risk stratification system, facilitating the distinction between benign and malignant adnexal neoplasms.

**Key Points:**

***Question*** How can a modified non-contrast O-RADS MRI scoring system be developed, and can this scoring system accurately classify adnexal masses?

***Findings*** The modified non-contrast O-RADS MRI system demonstrates excellent diagnostic performance and reliable risk stratification of adnexal masses by evaluating key imaging features.

***Critical relevance statement*** The modified non-contrast O-RADS MRI scoring system contributes to the risk stratification of adnexal masses, particularly for patients who cannot undergo contrast agent administration. This system provides a reliable tool to optimize treatment strategies and improve patient outcomes.

**Graphical Abstract:**

**Figure:**
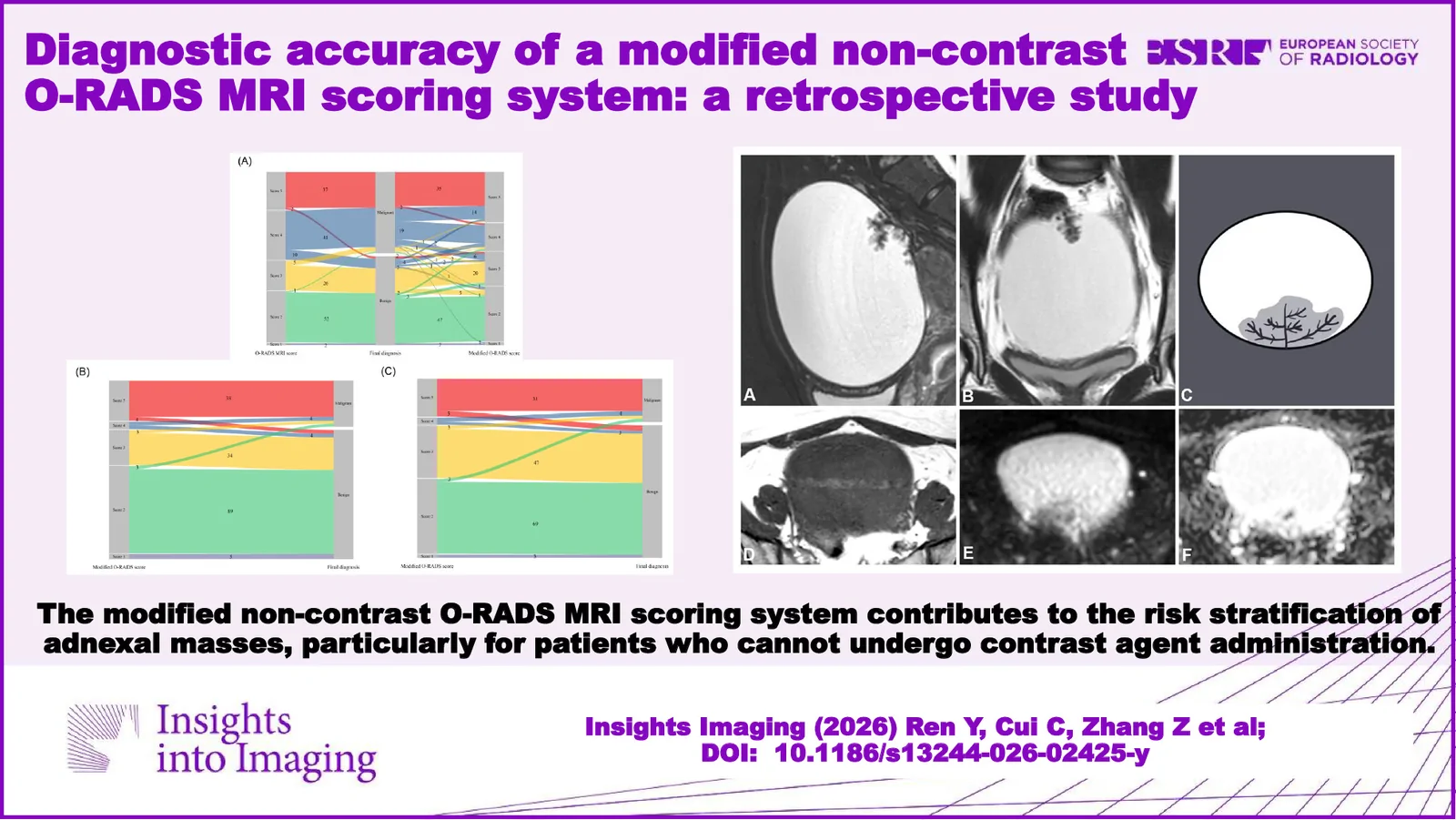

## Introduction

Adnexal masses are common disorders of the female reproductive system, and accurate preoperative evaluation is critical [1]. The majority of these lesions are benign, while malignant tumors are typically characterized by high invasiveness [2]. Therefore, it is necessary to establish a robust scoring system for early detection and distinguishing benign from malignant tumors in order to formulate appropriate treatment strategies and avoid unnecessary operations [3].

The Ovarian-Adnexal Reporting and Data System MRI Risk Stratification and Management System (O-RADS MRI) was developed based on the ADNEX magnetic resonance (MR) scoring system [4]. This system relies on the intravenous administration of gadolinium-based contrast agents to evaluate the time-intensity curve (TIC) pattern of dynamic contrast enhancement (DCE) in solid tissues [5]. Numerous studies have demonstrated that the O-RADS MRI scoring system possesses substantial diagnostic value in the grading and characterizing adnexal masses [6–9]. Nevertheless, the current O-RADS MRI protocol is time-consuming and costly, associated with potential risks of contrast allergies or renal dysfunction [10].

Consequently, we proposed a modified 5-point scoring system that eliminates the need for intravenous contrast agent administration. Several studies have indicated that diffusion-weighted imaging (DWI) can assist in distinguishing between benign and malignant adnexal masses [11, 12]. DWI reveals the microstructural characteristics of tissues by analyzing the Brownian motion of water molecules. Sahin et al [13] proposed a non-contrast O-RADS MRI scoring protocol, and the results demonstrated favorable diagnostic performance, whereas this study was deficient in effective quantitative indicators. The apparent diffusion coefficient (ADC) is a measurable parameter, and multiple studies have shown that ADC values of solid tissue play an important role in identifying tumor grades [12, 14]. However, misinterpreting the imaging features of solid components and insufficient reader experience compromise diagnostic accuracy [10]. Additionally, the previous study has indicated that approximately 80% of misclassified masses contain cystic components [9]. It is well known that malignant tumors typically exhibit high heterogeneity within solid tissue, associated with mixed tumor parenchyma, which increases interobserver variability in region of interest (ROI) placement, leading to inconsistent ADC measurements. In contrast, cystic components exhibit homogeneous signal intensity and well-defined margins, facilitating reliable and reproducible ROI positioning with clear target demarcation. Assouline et al [15] reported that the analysis of cystic components could enhance the diagnostic performance of the O-RADS MRI scoring system for adnexal masses, whereas the credibility may be suboptimal due to the absence of external validation. Thus, quantitative assessment of cystic components is critical for improving diagnostic accuracy.

Furthermore, although the O-RADS system identifies the morphology of solid components, it does not incorporate solid tissue morphology into risk stratification. Notably, several studies have highlighted that papillary projections exhibiting the “sea anemone sign” are characteristic features indicative of ovarian borderline tumors [16, 17]. This current research expands the scope of previous work on integrating the typical sign into the scoring system, with the aim of optimizing the risk categorization of adnexal masses.

Therefore, the present study aimed to investigate a practical modified non-contrast O-RADS MRI scoring system for noninvasive prediction of benign and malignant adnexal masses, as well as assessment of the diagnostic efficacy of risk stratifications.

## Materials and methods

### Ethics statement

The review boards of the two participating medical centers, including Tianjin First Central Hospital (Center I) and Jinhua Municipal Central Hospital (Center II), approved this multicenter study. Informed consent from patients was waived due to the retrospective nature of the study.

### Study design and population

We enrolled 142 female patients with 176 adnexal masses who underwent pelvic contrast-enhanced MRI examinations at Center I between January 2019 and January 2024. This group constituted Cohort A and was used to investigate key imaging signs. The validation set comprised 316 female patients with 352 adnexal lesions who underwent pelvic plain MRI examinations and were enrolled into the validation set during the period from January 2019 to June 2024. This set included two independent datasets: 164 patients (184 lesions) from Center I (Cohort B, internal validation) and 152 patients (168 lesions) from Center II (Cohort C, external validation). All patients underwent surgical intervention with histopathological diagnosis confirmation within the same healthcare system.

**Fig. 1:**
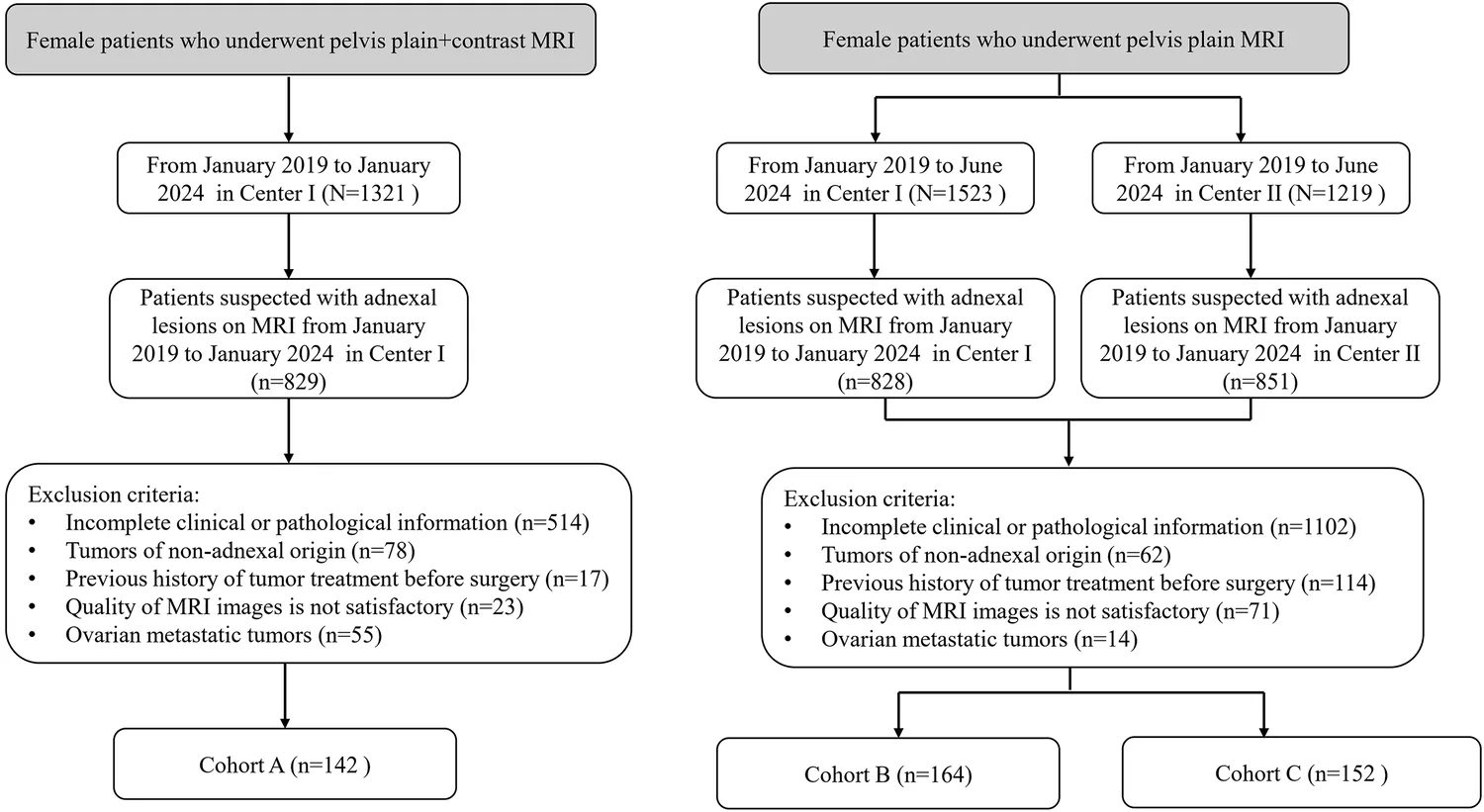
Flowchart of the study population

The inclusion criteria were as follows: (1) pelvic lesions suspected to originate from the adnexa on MRI images; (2) availability of general clinical data and histopathological results from surgical resection performed in the same institution; (3) no prior treatment for malignant adnexal lesions before the initial MRI examination; (4) MRI performed within 3 months before pathological diagnosis. Exclusion criteria were as follows: (1) tumors not located in the pelvic adnexal region on MRI images; (2) incomplete clinical or histopathological information; (3) history of tumor treatment before surgery; (4) suboptimal MRI image quality; (5) ovarian metastatic tumors. The patient selection flowchart is shown in Fig. 1.

### MRI protocol

The MRI examinations were conducted using 1.5-T and 3.0-T MRI scanners with body array coils at both centers. Intravenous injection of gadolinium diethylenetriaminepentaacetic acid (Gd-DTPA) contrast agent was performed at a dose of 0.2 mL/kg and a flow rate of 2–3 mL/s. Contrast-enhanced MRI was initiated at 30–40 s after contrast agent administration, with continuous scanning of the region of interest (ROI) covering the arterial, venous, and delayed phases. The detailed MRI scanning protocols are listed in the Supplementary Table S1.

### Image analysis

Two radiologists, with 3 and 6 years of experience in gynecological imaging diagnosis, have received training in the O-RADS MRI scoring system and independently evaluated the MRI features of the lesions, blinded to clinical and pathological data. For unilocular cystic masses or cystic-solid masses, three ROIs were selected in the cystic area to calculate the mean ADC value. For multilocular cystic masses, three ROIs were selected in each locule based on T2WI, and the average ADC value was measured. Each ROI was approximately 8 mm² avoiding any solid components. Besides, the clinical information should be recorded about age and menstrual status.

A modified non-contrast O-RADS MRI scoring system comprising five risk stratifications was developed, and a decision tree diagram was created to assist in lesion assessment. In the training dataset, the two radiologists assigned scores to each lesion using both the standard and modified non-contrast O-RADS MRI scoring systems, with a 3-month washout period. For the internal and external validation datasets, scores were assigned utilizing the modified O-RADS MRI scoring system. Each lesion was described separately in cases of multiple masses. Discrepancies were resolved through consensus.

### Reference standards

All patients enrolled in this study underwent surgical treatment, with postoperative pathological results serving as the gold standard. All specimens were examined by a pathologist experienced in adnexal lesions, who was blinded to clinical data and imaging findings, in accordance with the guidelines of the World Health Organization [18]. In this study, borderline tumors were classified as malignant tumors based on clinical management parallels.

### Statistical analysis

Statistical analyses were performed using SPSS (version 26.0) and MedCalc (version 20.022). Normality was assessed using the Shapiro–Wilk test. Continuous variables with normal distribution were described as mean ± standard deviation (mean ± SD), while non-normally distributed variables were expressed as median and interquartile range [Median (Q1, Q3)]. The independent samples *t*-test was applied for normally distributed continuous variables, and the Mann–Whitney U test was applied for non-normally distributed variables. Categorical data were presented as counts and percentages (%), and the chi-square test was used for comparison between two groups.

The final diagnosis was used as a binary variable, with borderline lesions included in the malignant group. Receiver operating characteristic (ROC) curve analysis was conducted to calculate the area under the curve (AUC) and determine the diagnostic cutoff values. With the optimal diagnostic cutoff value as the threshold, the diagnostic performance indicators were evaluated, including sensitivity, specificity, accuracy, positive predictive value (PPV), and negative predictive value (NPV). The DeLong test, net reclassification improvement (NRI), and integrated discrimination improvement (IDI) were performed to compare AUCs between the two scoring systems.

The interobserver agreements were evaluated using kappa (κ) statistics for categorical data and the intraclass correlation coefficient (ICC) for continuous variables. The agreements were classified as poor (0.00–0.40), moderate (0.41–0.60), good (0.61–0.80), and excellent (0.81–1.00) [19]. A *p*-value < 0.05 was considered statistically significant.

## Results

### Study population characteristics

In total, 458 female patients with 528 adnexal masses were included in the present study. Among these lesions, 355 were benign, and 173 were malignant. The training dataset comprised 142 patients (median age, 48 years [34–58 years]) from center I. The internal validation dataset involved 164 patients from center I (median age, 45 years [35–61 years]), and the external validation dataset enrolled 152 patients (median age, 47 years [IQR, 35–57 years]) from center II. Patients’ clinical characteristics are summarized in Supplementary Table S2. The results of the pathological diagnosis of the adnexal masses are listed in Supplementary Table S3.

### Image feature analysis

**Fig. 2:**
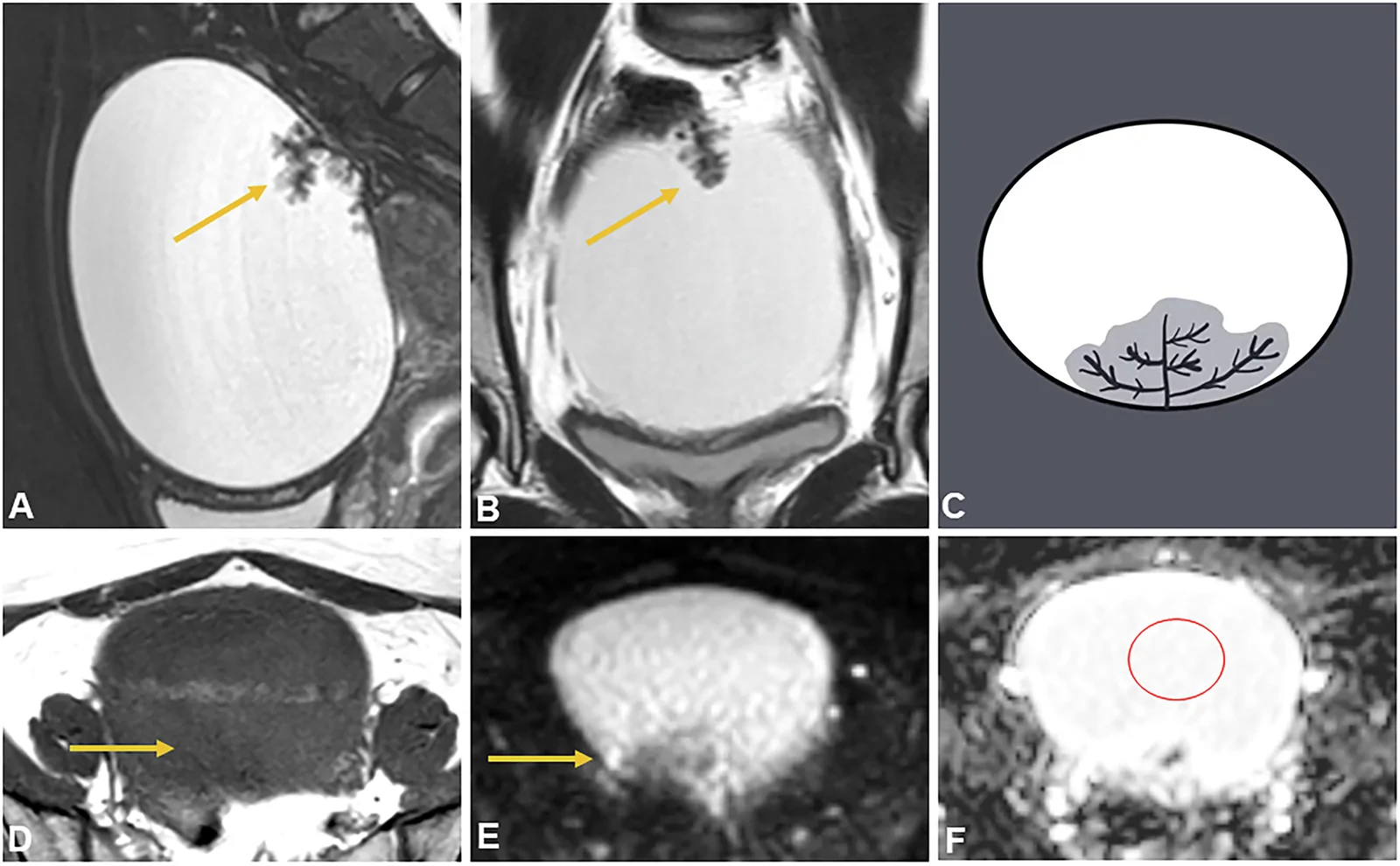
A 56-year-old woman with a left adnexal mass. The sagittal T2WI FS (**A**) and coronal T2WI (**B**) images showed cystic component with high intensity and the solid component with iso-low intensity (yellow arrow). The axial T1WI (**D**) image represented the cystic component with low intensity and the solid component with lower intensity (yellow arrow). The DWI (**E**) and ADC (**F**) maps demonstrated no obvious restricted diffusion of the solid component (yellow arrow). The ADC map showed an ADC mean value of 2.35 × 10^−^^3^ mm^2^/s in the cystic fluid (red circle). Corresponding schematic illustrations of “sea anemone sign” are shown at the right superior corner (**C**). This case was correctly categorized as malignant with a score of 4 due to “sea anemone-like” nodule and ADC value of cystic fluid in accordance with the modified non-contrast O-RADS MRI scoring system. The final pathology diagnosis confirmed that it was a left ovarian borderline mucinous cystadenoma

**Fig. 3:**
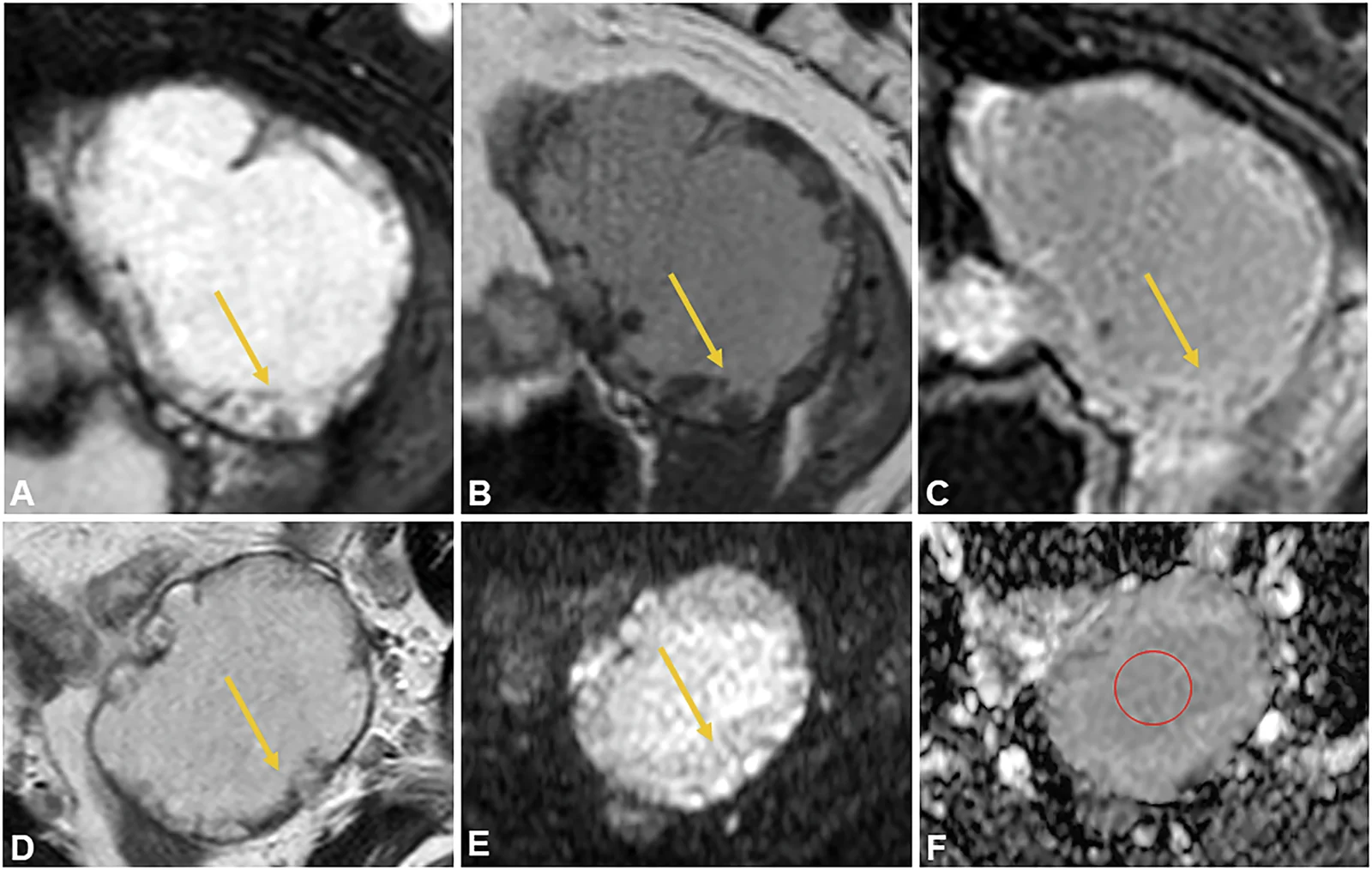
A 65-year-old woman presented with a solitary adnexal mass. The sagittal T2WI FS (**A**), axial T2WI (**D**) images showed the cystic component with high intensity and the solid component with iso-high intensity. The sagittal T1WI (**B**) image represented the cystic component with low intensity and the solid component with lower intensity. The sagittal T1WI enhanced image (**C**) represented enhancement of the solid component similar to myometrium. The DWI (**E**) and ADC (**F**) maps demonstrated no obvious limited diffusion of the solid component. The ADC map showed an ADC mean value of 1.60 × 10^−^^3^ mm^2^/s (red circle). This case was mistakenly classified as malignant with an O-RADS MRI score of 4, while correctly categorized as benign with a score of 3 due to no limited diffusion and ADC value of cystic fluid in accordance with the modified non-contrast O-RADS MRI scoring system. The final pathology diagnosis confirmed that it was a left ovarian seromucinous cystadenoma

In cohort A, the morphology of solid tissue demonstrated significant statistical differences in distinguishing benign from malignant lesions (*p* = 0.001). Notably, the sea anemone-like (SA) papillary projections were highly indicative of borderline lesions (*p* = 0.001) (Fig. 2). For cystic-solid lesions with non-sea-anemone-like (non-SA) papillary projections or irregular septa, an ADC value of cystic fluid > 1.74 × 10⁻³ mm²/s suggested a higher likelihood of malignancy (Fig. 3). The MRI characteristics are shown in Table 1.

**Table 1:** Comparison of imaging characteristics between benign and malignant adnexal masses in cohort A

Imaging characteristics	Cohort A		
	Benign masses (*n* = 92)	Malignant masses (*n* = 84)	*p*-value
Peritoneal, mesenteric, or omental nodularity or irregular thickening	0 (0)	2 (2.4)	0.000
Lipid component	17 (18.5)	0 (0)	0.002
Solid component morphological feature	22 (23.9)	76 (90.5)	0.001
Papillary projection			
Non-SA papillary projection	0 (0.0)	7 (83.3)	
SA papillary projection	0 (0.0)	18 (21.4)	0.001
Mural nodule	6 (6.5)	12 (14.3)	
Irregular septum	8 (8.7)	6 (7.1)	
Solid lesion	5 (5.4)	5 (6.0)	
Other type	3 (3.3)	28 (33.3)	
Solid component DWI signal			0.001
Hypointensity	7 (7.6)	3 (3.6)	
Isointensity	6 (6.5)	7 (8.3)	
Hyperintensity	9 (9.8)	66 (78.6)	
Solid component ADC signal			0.001
Hypointensity	14 (15.2)	73 (86.9)	
Isointensity	1 (1.1)	1 (1.2)	
Hyperintensity	7 (7.6)	2 (2.4)	
Solid component restricted diffusion	9 (9.8)	65 (77.4)	0.001
Unilocular/multilocular	29/26	4/2	0.678
Cystic fluid type			0.871
Pure liquid	31 (33.7)	4 (4.8)	
Endometriotic liquid	14 (15.2)	1 (1.2)	
Proteinaceous and hemorrhagic fluid	10 (10.9)	1 (1.2)	
Cystic fluid ADC value of cystic-solid mass			0.029
> 1.74 (× 10^−3^ mm^2^/s)	18 (19.6)	66 (78.6)	
≤ 1.74 (× 10^−^^3^ mm^2^/s)	12 (13.0)	4 (4.8)	

The data are presented as numbers, with percentages in parentheses

*DWI* diffusion-weighted imaging, *ADC* apparent diffusion coefficient, *Non-SA papillary projection* non-sea-anemone-like papillary projection, *SA papillary projection* sea anemone-like papillary projection

In cohorts B and C, all lesions assigned a score of 5 exhibited restricted diffusion in the solid components. 69% and 72.2% of the score 5 lesions showed ADC values of cystic fluid > 1.74 × 10⁻³ mm²/s, respectively. The frequency distributions of modified non-contrast O-RADS categories for adnexal masses across the two validation sets are presented in Table 2.

**Table 2:** Frequency distributions of modified non-contrast O-RADS MRI scores for adnexal masses in cohorts B and C

Imaging characteristics	Cohort B				Cohort C			
	2 (*n* = 92)	3 (*n* = 37)	4 (*n* = 8)	5 (*n* = 42)	2 (*n* = 72)	3 (*n* = 50)	4 (*n* = 7)	5 (*n* = 36)
Peritoneal, mesenteric, or omental nodularity or irregular thickening	0 (0)	0 (0)	0 (0)	10 (23.8)	0 (0)	0 (0)	0 (0)	5 (13.9)
Lipid component	24 (26.1)	0 (0)	0 (0)	0 (0)	24 (33.3)	0 (0)	0 (0)	0 (0)
Solid component morphological feature								
Papillary projection								
Non-SA papillary projection	0 (0)	0 (0)	2 (25)	0 (0)	1 (1.4)	1 (2)	2 (28.6)	0 (0)
SA papillary projection	0 (0)	0 (0)	2 (25)	0 (0)	0 (0)	0 (0)	1 (14.3)	0 (0)
Mural nodule	0 (0)	2 (5.4)	0 (0)	6 (14.3)	1 (1.4)	1 (2)	0 (0)	11 (30.6)
Irregular septum	0 (0)	8 (21.6)	4 (50)	0 (0)	1 (1.4)	4 (8)	4 (57.1)	0 (0)
Solid lesion	5 (5.4)	3 (8.1)	0 (0)	8 (19)	3 (4.2)	4 (8)	0 (0)	9 (25)
Other type	2 (2.2)	2 (5.4)	0 (0)	28 (66.7)	1 (1.4)	6 (12)	0 (0)	16 (44.4)
Solid component DWI signal								
Hypointensity	5 (5.4)	2 (5.4)	0 (0)	0 (0)	5 (6.9)	4 (8)	0 (0)	0 (0)
Isointensity	2 (2.2)	9 (24.3)	2 (25)	0 (0)	1 (1.4)	9 (18)	1 (14.3)	0 (0)
Hyperintensity	0 (0)	4 (10.8)	6 (75)	42 (100)	1 (1.4)	3 (6)	6 (85.7)	36 (100)
Solid component ADC signal								
Hypointensity	5 (5.4)	8 (21.6)	6 (75)	42 (100)	5 (6.9)	10 (20)	6 (85.7)	36 (100)
Isointensity	2 (2.2)	6 (16.2)	2 (25)	0 (0)	1 (1.4)	3 (6)	1 (14.3)	0 (0)
Hyperintensity	0 (0)	1 (2.7)	0 (0)	0 (0)	1 (1.4)	3 (6)	0 (0)	0 (0)
Solid component restricted diffusion								
Yes	0 (0)	0 (0)	6 (75)	42 (100)	0 (0)	0 (0)	6 (85.7)	36 (100)
No	7 (7.6)	15 (40.5)	2 (25)	0 (0)	7 (9.7)	16 (32)	1 (14.3)	0 (0)
Unilocular/multilocular								
Unilocular	44 (47.8)	4 (10.8)	0 (0)	0 (0)	32 (44.4)	3 (6)	0 (0)	0 (0)
Multilocular	17 (18.5)	18 (48.6)	0 (0)	0 (0)	9 (12.5)	31 (62)	0 (0)	0 (0)
Cystic fluid type								
Pure liquid	19 (20.7)	17 (45.9)	0 (0)	0 (0)	10 (13.9)	22 (44)	0 (0)	0 (0)
Endometriotic liquid	42 (45.7)	0 (0)	0 (0)	0 (0)	31 (43.1)	0 (0)	0 (0)	0 (0)
Proteinaceous/hemorrhagic fluid	0 (0)	5 (13.5)	0 (0)	0 (0)	0 (0)	12 (24)	0 (0)	0 (0)
Cystic fluid ADC value								
> 1.74 (× 10^−^^3^ mm^2^/s)	7 (7.6)	10 (27)	3 (37.5)	29 (69)	27 (37.5)	42 (84)	6 (85.7)	26 (72.2)
≤ 1.74 (× 10^−^^3^ mm^2^/s)	46 (50)	2 (5.4)	1 (12.5)	3 (7.1)	42 (58.3)	4 (8)	0 (0)	3 (8.3)

The data are presented as numbers, with percentages in parentheses

*DWI* diffusion-weighted imaging, *ADC* apparent diffusion coefficient, *Non-SA papillary projection* non-sea-anemone-like papillary projection, *SA papillary projection* sea anemone-like papillary projection

### Establishment of the modified non-contrast O-RADS MRI scoring system

**Fig. 4:**
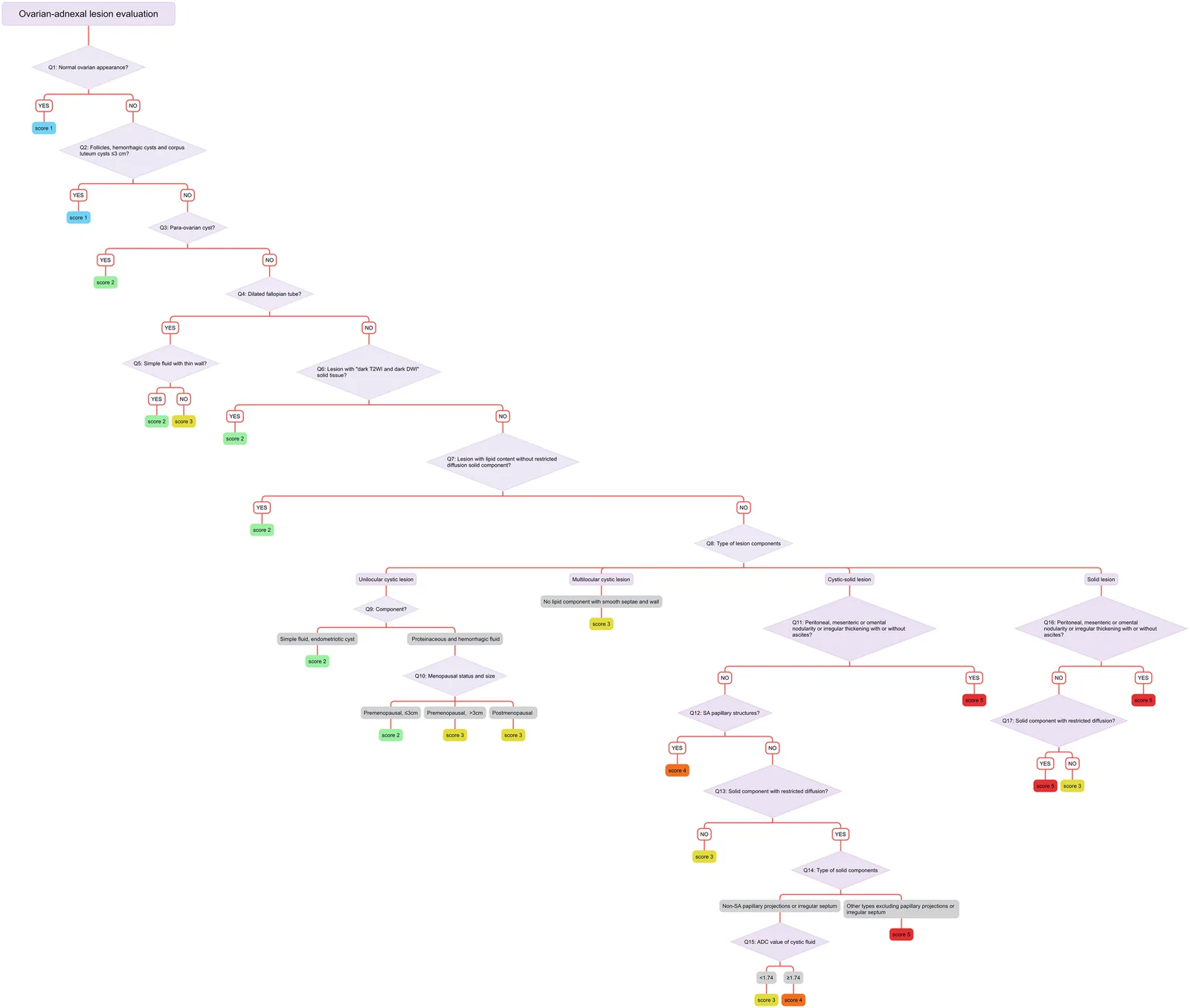
The decision tree diagram of the modified non-contrast O-RADS MRI scoring system

A modified non-contrast O-RADS MRI scoring system (Table 3) was developed based on a comprehensive assessment of plain MRI features in the training set and the standard O-RADS MRI scoring system. To ensure an accurate O-RADS score for each adnexal mass during evaluation, a decision tree diagram was designed to guide the observers to analyze the characteristics step by step (Fig. 4 and Supplementary Table S4).

**Table 3:** Modified non-contrast O-RADS scoring system

Modified non-contrast O-RADS score	Risk category	MRI image features
1	Normal ovaries or physiologic appearances	No ovarian lesion
		Follicles, hemorrhagic cysts, and corpus luteum cysts ≤ 3 cm
2	Almost certainly benign	Unilocular cystic lesion: simple fluid, endometriotic cyst
		Unilocular cystic lesion: ≤ 3 cm with proteinaceous and hemorrhagic fluid in a premenopausal woman
		Lesion with lipid content and solid component without restricted diffusion
		Lesion with “dark T2WI and dark DWI” solid tissue
		Dilated fallopian tube fluid: simple fluid with thin wall
		Para-ovarian cyst
3	Predominantly benign	Unilocular cystic lesion: > 3 cm with proteinaceous and hemorrhagic fluid in a premenopausal woman
		Unilocular cystic lesion: proteinaceous and hemorrhagic fluid in a postmenopausal woman
		Multilocular cyst: no lipid component with smooth septae
		Cystic-solid mass with solid components (excluding SA papillary projections) no restricted diffusion
		Cystic-solid mass with solid components (non-SA papillary projections or irregular septum) restricted diffusion, and cystic fluid ADC value < 1.74 (× 10^−^^3^ mm^2^/s)
		Dilated fallopian tube: non-simple fluid or simple fluid with thick wall
4	Likely to be malignant	SA papillary projections
		Cystic-solid mass with solid components (non-SA papillary projections or irregular septum) restricted diffusion, and cystic fluid ADC value ≥ 1.74 (× 10^−^^3^ mm^2^/s)
5	Highly suspicious for malignancy	Solid mass or cystic-solid mass with solid components (other than papillary projections or irregular septum) restricted diffusion
		Peritoneal, mesenteric or omental nodularity or irregular thickening with or without ascites

*O-RADS* Ovarian-Adnexal Reporting and Data System, *MRI* magnetic resonance imaging, *T2WI* T2-weighted imaging, *DWI* diffusion-weighted imaging, *ADC* apparent diffusion coefficient. *Non-SA papillary projection* non-sea-anemone-like papillary projection, *SA papillary projection* sea anemone-like papillary projection

### Diagnostic results of the O-RADS MRI and the modified non-contrast O-RADS MRI scoring system

The distributions of the O-RADS MRI scores and the modified O-RADS scores of the adnexal masses are listed in Table 4. Among the 51 adnexal masses classified as score 4, 41 (80.4%) were malignant and 10 (19.6%) were benign. Among the 39 adnexal masses given the score of 5, 37 (94.9%) were malignant and 2 (5.1%) were benign.

**Table 4:** Frequency distributions of adnexal masses in all groups stratified by standard O-RADS and modified non-contrast O-RADS MRI scoring system

Score categories	Score 1		Score 2		Score 3		Score 4		Score 5	
	Benign	Malignant	Benign	Malignant	Benign	Malignant	Benign	Malignant	Benign	Malignant
Cohort A with O-RADS MRI score	2	0	52	1	26	5	10	41	2	37
Cohort A with modified O-RADS score	2	1	54	3	27	8	7	22	2	50
Cohort B with modified O-RADS score	5	0	89	3	34	3	4	4	4	38
Cohort C with modified O-RADS score	3	0	69	3	47	3	3	4	5	31

*O-RADS* Ovarian-Adnexal Reporting and Data System, *MRI* magnetic resonance imaging

In the training set, using the basis of the modified non-contrast O-RADS MRI scoring system, the proportions of benign masses among lesions scored as 1, 2, and 3 were 66.7%, 94.7%, and 77.1%, respectively. For lesions rated as score 4 and score 5, the proportions of malignant neoplasms were 75.9% and 96.1%, respectively.

**Fig. 5:**
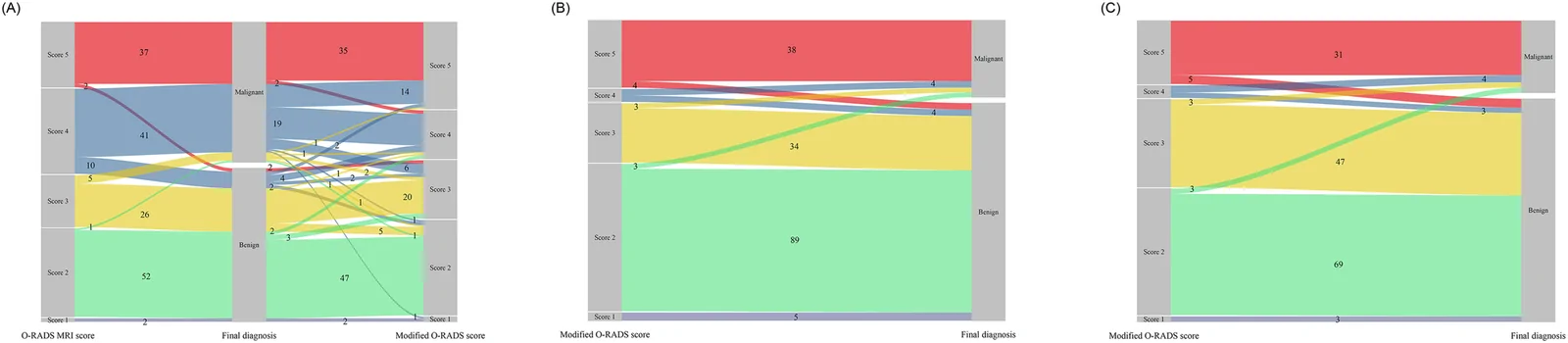
**A** The Sankey diagram visualized correspondence and differences between the O-RADS MRI scoring system and the modified non-contrast O-RADS MRI scoring system based on the final diagnosis in cohort A. **B** The Sankey diagram demonstrated the correspondence between the modified non-contrast O-RADS MRI scoring system and final diagnosis in cohort B. **C** The Sankey diagram presented the correspondence between the modified non-contrast O-RADS MRI scoring system and final diagnosis in cohort C

In cohorts B and C, 89 and 69 benign masses were classified as score 2, accounting for 96.7% and 95.8%, respectively. 38 and 31 malignant lesions were assigned score 5, constituting 90.5% and 86.1% of the respective sets. The distributions of the two scoring systems over all datasets are shown in Fig. 5.

### Diagnostic performance of the O-RADS MRI and the modified non-contrast O-RADS MRI scoring system

**Fig. 6:**
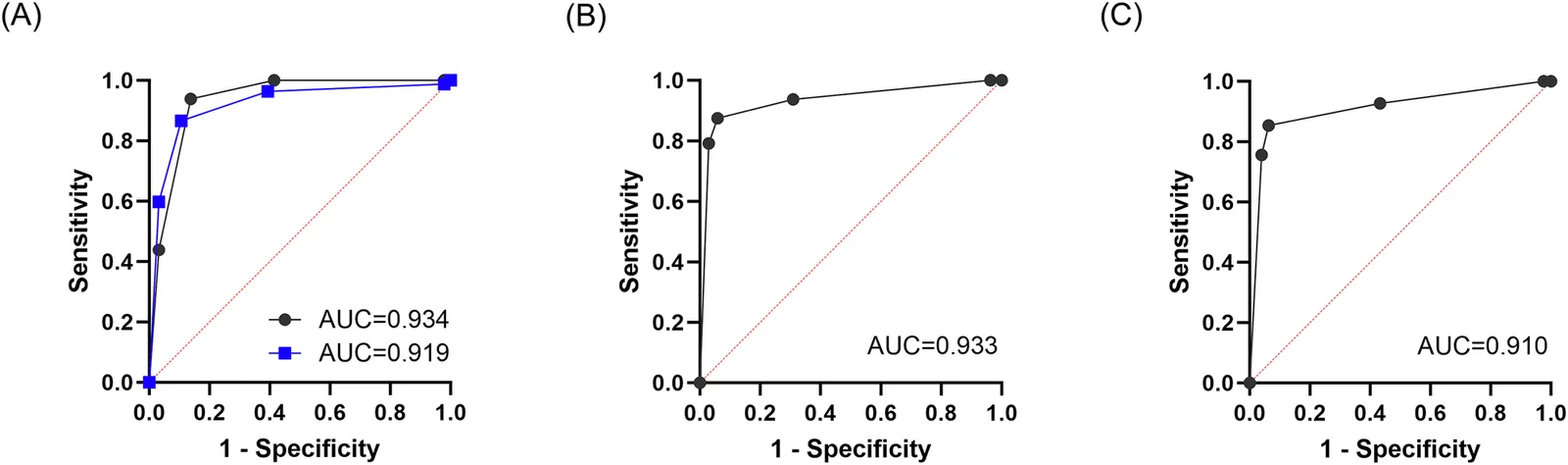
Receiver operating characteristic curves (ROC) of the diagnostic performance of the O-RADS MRI risk stratification system for differentiating benign and malignant adnexal masses. **A** ROC curve and AUC in cohort A with the O-RADS MRI (black circle) and modified non-contrast O-RADS MRI scoring system (blue square). **B** The ROC curve and AUC of the modified non-contrast O-RADS MRI scoring system in cohort B. **C** The ROC curve and AUC of the modified non-contrast O-RADS MRI scoring system in cohort C

In cohort A, the AUC of the O-RADS MRI scoring system for predicting the final diagnosis was slightly higher than that of the modified scoring system with no significant statistical differences (0.934 vs. 0.919, *p* > 0.05). For the modified O-RADS MRI scoring system in cohort A, the optimal diagnostic cutoff value for predicting malignancy was determined as a score of ≥ 4, which yielded a sensitivity of 86.6%, specificity of 89.4%, PPV of 87.7%, and NPV of 88.4%. The DeLong test showed no statistically significant difference in AUC between the two systems (Z = 0.690, *p* = 0.49, 95% CI [−0.027 to 0.057]). Compared with the O-RADS MRI scoring system, the NRI and IDI of the modified non-contrast O-RADS MRI scoring system obtained as −4.13% (*p* = 0.403, 95% CI [−13.79% to 5.54%]) and −0.04 (*p* = 0.405, 95% CI [−0.14 to 0.06]), respectively. In cohorts B and C, the AUCs were 0.933 and 0.910, respectively (Table 5). The ROC curves of the two scoring systems are displayed in Fig. 6.

**Table 5:** Diagnostic performance of standard O-RADS and modified non-contrast O-RADS categories in distinguishing benign and malignant adnexal masses

Parameters	O-RADS MRI score in Cohort A	Modified non-contrast O-RADS MRI score in Cohort A	Modified non-contrast O-RADS MRI score in Cohort B	Modified non-contrast O-RADS MRI score in Cohort C
AUC	0.934 (0.896–0.971)	0.919 (0.874–0.963)	0.933 (0.884–0.983)	0.910 (0.848–0.973)
Accuracy (%)	89.8	88.1	92.4	91.7
Sensitivity (%)	93.9 (88.7–99.1)	86.6 (79.2–94.0)	87.5 (78.1–96.9)	85.4 (74.6–96.2)
Specificity (%)	86.2 (79.1–93.2)	89.4 (83.1–95.6)	94.1 (90.2–98.1)	93.7 (89.5–97.9)
PPV (%)	85.6 (78.2–92.9)	87.7 (80.5–94.9)	84.0 (73.9–94.2)	81.4 (69.7–93.1)
NPV (%)	94.2 (89.2–99.1)	88.4 (82.0–95.6)	95.5 (92.0–99.0)	95.2 (91.4–98.9)

Data in parentheses are 95% confidence interval

*O-RADS* Ovarian-Adnexal Reporting and Data System, *MRI* magnetic resonance imaging, *AUC* area under the curve, *PPV* positive predictive value, *NPV* negative predictive value

### Failure analysis

For clinical management purposes, we adopted a binary classification approach: scores 1–3 were categorized as benign or likely benign, and scores 4–5 were categorized as malignant or highly suspicious for malignancy. Based on this binary stratification, the modified scoring system appropriately reclassified 8 lesions that had been misclassified by the O-RADS MRI scoring system. These included serous cystadenoma (2 cases), seromucinous cystadenoma (1 case), borderline mucinous tumors (2 cases), mature cystic teratoma (1 case), and endometriomas with wall nodules (2 cases). Conversely, 11 lesions correctly classified by the O-RADS MRI scoring system were misclassified by the modified scoring system, composed of borderline serous tumors (2 cases), borderline mucinous tumors (4 cases), borderline seromucinous tumors (3 cases), mature cystic teratoma (1 case), and endometrioid adenocarcinoma (1 case).

### Evaluation of inter-reader agreement

The modified non-contrast O-RADS MRI score showed excellent к values in all sets. The ADC values of cystic fluid showed good interobserver consistency with ICC of 0.713 (95% CI 0.479–0.852). Regarding the descriptions of the imaging features, inter-reader consistency for the lesion category was good to excellent (Supplementary Table S5).

## Discussion

This multicenter study, incorporating internal and external validations, aimed to develop a modified non-contrast O-RADS MRI scoring system. The findings highlight that the modified 5-point scoring system, which integrates morphological assessment, image signal analysis, and quantitative parameters, exhibits a diagnostic efficacy comparable to that of the conventional O-RADS MRI scoring system. This modified scoring system ensures comprehensive and accurate categorization of adnexal masses, thereby providing a reliable basis for formulating optimal clinical treatment strategies.

The standard O-RADS MRI scoring system relies on intravenous injection of contrast agents to monitor lesion enhancement patterns [5, 20]. However, this approach has certain limitations in clinical application, which may trigger severe allergic reactions or increase the risk of renal impairment [21, 22]. Besides, some hospitals lack the resources to perform dynamic contrast-enhanced MR scanning [23], which accounts for the limitations in equipment or medical resources, reducing the effectiveness of the O-RADS system [10]. Our results indicate that the modified O-RADS scoring system retains considerable diagnostic value while shortening the scanning time and avoiding contrast-associated risks. This has significant implications for primary healthcare facilities where the implementation of contrast-enhanced sequences is not feasible. Therefore, it is crucial to establish a practical scoring system independent of contrast agents for adnexal lesions evaluation.

Previous studies have confirmed that the DWI sequence plays a vital role in diagnosing ovarian lesions [12, 24]. Most research exploring the combined application of DWI and the O-RADS MRI scoring system has focused on restricted diffusion in solid components [14, 25]. In cohort A of this study, among cystic-solid and solid lesions, those with restricted diffusion of solid components accounted for 9.8% and 78.6% in the benign and malignant groups, respectively, with a statistically significant difference. In cohorts B and C, all the lesions scored as 5 exhibited restricted diffusion in solid tissue. These results are consistent with those of previous studies. Hence, the degree of diffusion restriction in solid components may potentially contribute to the classification of ovarian lesions.

The standard O-RADS MRI system mainly relies on the solid component manifestations rather than cystic fluid characteristics. The research by Naggara et al [9] pointed out that majority of misclassifications were caused by the misinterpretation of solid tissues and time-intensity curve (TIC), leading to incorrect risk assignment and distorted clinical diagnosis. In clinical practice, solid components exhibit high heterogeneity, and consistency of ADC value measurement among different observers is often controversial, which is attributed to differences in the selection of ROIs. Assouline et al [15] demonstrated that an ADC value below 1.69 × 10⁻³ mm²/s for cystic fluid carried an almost negligible risk of malignancy in cystic-solid lesions. In our study, there was a statistically significant difference in the ADC value of cyst fluid between benign and malignant groups, with an optimal threshold of 1.74, which roughly coincides with the findings of Assouline et al’s research. It can be inferred that malignant tumors are primarily characterized by epithelial tumors with a serous component, where water molecules do not show restricted diffusion. On the contrary, the majority of benign lesions contain endometriotic fluid, hemorrhagic components, or protein-rich fluid, which strongly impedes the free diffusion of water molecules, resulting in lower measured ADC values of cyst fluid [15]. Above all, the ADC value of cyst fluid was integrated into the modified O-RADS MRI scoring system, which attributed to boosting the diagnostic effectiveness in evaluating cystic and cystic-solid lesions.

Serous tumors are the most common type of borderline ovarian neoplasms [26], accounting for 50–70% of all borderline tumors [27]. Regardless of the fact that the biological behavior exhibits relatively indolent with a favorable prognosis generally, borderline tumors possess the potential for malignancy. Fertility-sparing surgery is the first-line treatment option for patients desiring fertility preservation [28]. Tanaka et al [17] defined the typical sign of “sea anemone-like” papillary structure as hypointense branch-like projections inside on T2WI, which is characteristically composed of numerous dilated glands in a papillary pattern with mucus secretion and containing fibrous structures [29]. A number of articles have highlighted that this typical morphology is the critical feature of the borderline ovarian tumors [16, 17]. In our research, lesions presenting as SA papillary projections were mostly categorized as score 4 and verified as borderline tumors, consistent with previous studies. It should be noted that incorporating borderline ovarian tumors into the malignant group may reduce the specificity of our model, as these tumors typically show lower cellularity, less restricted diffusion, and overlapping imaging features with benign solid lesions relative to carcinomas, yet are classified as malignant in the analysis. This inherent overlap in imaging features tends to reduce diagnostic specificity and accuracy. Nevertheless, this grouping strategy helps maintain high sensitivity for detecting the lesions requiring necessary clinical intervention. Accordingly, characteristic morphological features were integrated into the modified scoring system to improve the identification of serous borderline tumors.

This study showed that the AUC of the modified scoring system was comparable to that of the standard scoring system. Although the NRI and IDI of the two scoring systems were both negative, the differences were not statistically significant. These results indicate that the non-contrast O-RADS protocol achieves equivalent diagnostic accuracy relative to the standard scoring system, with improved clinical applicability and safety in patients unfit for contrast-enhanced MRI. Sahin et al [13] proposed a non-contrast O-RADS scoring protocol, demonstrating its significant diagnostic value. However, the study only used single-center data and lacked validation results, leading to lower stability and reliability of the simplified protocol. Our study employed multicenter samples with two independent validation datasets, and both reflected strong credibility and reproducibility [30]. This implies that the modified scoring system holds significant value under circumstances where patients are unsuitable for performing an enhanced scan. In the present study, the interobserver agreement for the modified O-RADS score across all cohorts obtained excellent Kappa values, indicating good reliability and reproducibility. The research by Akkaya et al [31] also illustrated that the interobserver consistency was high among three physicians. Furthermore, Cabedo et al [32] designed a checklist to help obtain appropriate O-RADS scores regardless of reader experience. In this study, we created a decision tree diagram to guide the radiologists in analyzing these MRI signs step by step. The agreements in the evaluation of these MRI features reached good to excellent consistency, suggesting that these signs were highly objective and could be accurately identified by observers with various levels of experience.

There were some cases misclassified by both scoring systems. On the one hand, lesions misclassified by the standard scoring protocol were appropriately scored by the modified system. We reasoned that the enhancement degree of solid tissue in some benign neoplasms was usually lower than that of the uterine myometrium, leading to their incorrect classification as score 4 by the standard protocol. In contrast, these lesions could be properly categorized as score 3 based on the absence of restricted diffusion in solid components in the modified scoring model. On the other hand, several cases, mainly borderline epithelial tumors, were miscategorized as benign by the modified scoring system, because the solid component did not show restricted diffusion on DWI. Furthermore, although borderline tumors tended to exhibit benign appearances, their potential malignant nature could not be ignored.

There are several limitations in the current study. First, it is a retrospective study that is susceptible to selection bias, and prospective research is needed to enhance its reliability in future investigations. Second, the sample size is relatively small. Further investigations with an expanded network of centers and larger sample sizes are imperative to confirm diagnostic effectiveness. Third, external validation was conducted by the same readers rather than independent observers across institutions, which may restrict its interobserver reliability and generalizability. Further multicenter studies with independent readers are warranted to verify interobserver agreement. In addition, ADC values may be affected by factors such as MRI scanner type, magnetic field strength, and image acquisition parameters. Since the ADC threshold in this study was derived from retrospective analyses, it may not be directly generalizable across diverse equipment and scanning protocols. Therefore, future prospective studies with standardized ADC acquisition are required to further validate the robustness and generalizability of these findings across different MRI systems.

## Conclusion

In conclusion, the modified non-contrast O-RADS MRI scoring system exhibited high diagnostic accuracy and facilitated the differentiation of malignant from benign neoplasms in patients unable to undergo contrast administration. The scoring system incorporates typical solid component morphology, the degree of restricted diffusion, and the ADC value of cystic fluid, which are critical for accurate O-RADS score assignment. Therefore, this modified scoring system may serve as a valuable and noninvasive tool for the risk stratification of adnexal masses.

## Supplementary information

ELECTRONIC SUPPLEMENTARY MATERIAL

## Data Availability

Data sharing is not applicable to this article as no datasets were generated or analyzed during the current study.

## Abbreviations

ADC: Apparent diffusion coefficient
AUC: Area under the curve
DWI: Diffusion-weighted imaging
IDI: Integrated discrimination improvement
MR(I): Magnetic resonance (imaging)
NPV: Negative predictive value
NRI: Net reclassification improvement
O-RADS: Ovarian-Adnexal Reporting and Data System
PPV: Positive predictive value
ROC: Receiver operating characteristic
ROI: Region of interest
