# Supplementary material for: Diagnostic accuracy of a modified non-contrast O-RADS MRI scoring system: a retrospective study
Source: Insights Imaging. 2026 Oct 16;17:261. doi: 10.1186/s13244-026-02425-y (PMC13675434; doi:10.1186/s13244-026-02425-y)
Supplement: Supplementary file 1 [file 13244_2026_2425_MOESM1_ESM.pdf]

Diagnostic accuracy of a modified non-contrast O-RADS MRI scoring system: a retrospective study

ELECTRONIC SUPPLEMENTARY MATERIAL

Supplementary Table S1. MRI scanning protocol parameters

Parameters	Philips Ingenia 3.0T MR (Center I)				Siemens Trio Tim 3.0T MR (Center I)				Siemens Avanto 1.5T MR (Center II)		
Sequence	T2WI-TSE<br>T2WI-FS	T1WI-DIXON-TSE	DWI-EPI	T1WI-vibe-twist-DIXON	T2WI-TSE	T2WI-FS	T1WI-TSE<br>T1WI-FS	DWI-EPI	T2WI-FSE<br>T2WI-FS	T1WI-TSE	DWI-EPI
Direction	Axial<br>Coronal<br>Sagittal	Axial<br>Sagittal	Axial	Axial	Axial	Sagittal<br>Coronal	Axial	Axial	Axial<br>Coronal<br>Sagittal	Axial	Axial
TR (ms)	2968-4934	624	6000	4.2	3600	4271-4700	709-768	5600	3100-4800	531	6928
TE (ms)	90-100	7	82	2.0	98	88-90	11-12	81	81-95	11	87
FOV (mm^2)	452×452	446×446	400×400	300×300	240×240	240×240	240×240	280×224	270×270	270×270	280×280
Thickness （mm）	3.0-4.0	4.0	4.0	3.0	3.0	4.0	4.0	4.0	3.0-4.0	4.0	4.0
Slice interval （mm）	1.0	1.0	1.0	2.5	0.4	0.4-0.5	0.4	0.4	0.4-0.5	0.4-0.5	0.4-0.5
b value	-	-	0,1000	-	-	-	-	0,1000	-	-	0,1000

TR=Repetition Time, TE= Echo Time, FOV= Field of View, T2WI-TSE= T2-weighted imaging-turbo spin echo, T2WI-FS= T2 weighted imaging-fat suppression, T1WI-DIXON-TSE = T1-weighted imaging- DIXON water fat separation sequence-turbo spin echo, DWI-EPI= diffusion weighted imaging-echo planar imaging, T1WI-vibe-twist-DIXON= T1 weighted imaging-vibe-twist-DIXON water fat separation sequence-turbo spin echo, T1WI-TSE= T1-weighted imaging-turbo spin echo, T1WI-FS= T1-weighted imaging- fat suppression, T2WI-FSE= T2-weighted imaging-fast spin echo

Supplementary Table S2. Characteristics of the study population

	Training set			Internal validation set			External validation set		
	**Benign (n=74)**	**Malignant (n=68)**	***P* value**	**Benign (n=125)**	**Malignant (n=39)**	***P* value**	**Benign (n=114)**	**Malignant (n=38)**	***P* value**
Age	42.9 (±12.5)	48.99 (±12.2)	0.004	40 (31, 57)	54 (48, 64)	0.000	42 (33,59)	54 (47,63)	0.003
Menstrual Status			0.003			0.010			0.004
Postmenopausal	21 (28.4)	36 (52.9)		36 (28.8)	20 (51.3)		39 (34.2)	23 (60.5)	
Premenopausal	53 (71.6)	32 (47.1)		89 (71.2)	19 (48.7)		75 (65.8)	15 (39.5)	
Maximum Diameter (cm)	5.8 (3.5,9.9)	9.4 (6.6,13.3)	0.000	6.8 (4.9,10.1)	9.3 (6.5,14.2)	0.010	6.7 (4.8,9.9)	7.7 (4.7,11.2)	0.545
Laterality			0.937			0.003			1.000
Unilateral	57 (77.0)	52 (76.5)		115 (92.0)	29 (74.4)		102 (89.5)	34 (89.5)	
Bilateral	17 (23.0)	16 (23.5)		10 (8)	10 (25.6)		12 (10.5）	4 (10.5)	

Values are mean ± standard deviation, count (%), or median (Q1, Q3).

Supplementary Table S3. Histopathology diagnosis of the adnexal masses for the whole training and validation set

Pathology diagnosis	Training set	Internal validation set	External validation set
**Benign tumor**	**92 (52.27)**	**136 (73.91)**	**127 (75.59)**
Endometriotic cyst	17 (9.66)	40 (21.73)	31 (18.45)
Cystic/mature teratoma	20 (11.36)	33 (17.93)	31 (18.45)
Serous cystadenoma	17 (9.66)	18 (9.78)	13 (7.73)
Mucinous cystadenoma	9 (5.11)	14 (7.60)	20 (11.90)
Seromucinous cystadenoma	5 (2.84)	6 (3.26)	6 (3.57)
Cystadenofibroma	8 (4.55)	2 (1.08)	4 (2.38)
Fibroma/ cystadenoma	6 (3.41)	8 (4.34)	15 (8.92)
Benign Brenner tumor	0 (0)	2(1.08)	0 (0)
Leydig cell tumor	0 (0)	0 (0)	1 (00.59)
Endometrioid adenofibroma	1 (0.57)	0 (0)	0 (0)
Corpus luteum cyst/simple cyst/follicular cyst	9 (5.11)	13 (7.06)	6 (3.57)
**Malignant/borderline tumor**	**84 (47.73)**	**48 (26.08)**	**41 (24.40)**
Serous borderline tumor	21 (11.93)	4 (2.17)	2 (1.19)
Mucinous borderline tumor	7 (3.98)	1 (00.54)	2 (1.19)
Borderline endometrioid cystadenoma	0 (0)	0 (0)	1 (00.59)
Seromucinous borderline tumor	8 (4.55)	0 (0)	0 (0)
Serous-endometrioid mixed carcinoma	0 (0)	1 (00.54)	0 (0)
Serous carcinoma	24 (13.64)	28 (15.21)	20 (11.90)
Mucinous carcinoma	3 (1.70)	2 (1.08)	2 (1.19)
Clear cell carcinoma	12 (6.82)	7 (3.80)	4 (2.38)
Endometrioid adenocarcinoma	7 (3.98)	2 (1.08)	2 (1.19)

Seromucinous carcinoma	0 (0)	0 (0)	2 (1.19)
Sertoli-Leydig cell tumor	0 (0)	0 (0)	1 (00.59)
Dysgerminoma	0 (0)	1 (00.54)	0 (0)
Granulosa cell tumor	0 (0)	2 (1.08)	4 (2.38)
Yolk sac tumor	1 (0.57)	0 (0)	0 (0)
Malignant transformation of cystic teratoma	1 (0.57)	0 (0)	0 (0)
Gastrointestinal stromal tumor (GIST)	0 (0)	0 (0)	1 (00.59)

Values are shown with count (%).

Supplementary Table S4. The questions in the decision tree diagram

Item	Evaluation question
Q1	Is the ovarian appearance normal?
Q2	Are follicles, hemorrhagic cysts, and corpus luteum cysts ≤3 cm in size?
Q3	Is this lesion a para-ovarian cyst?
Q4	Is there any dilated fallopian tube present?
Q5	Is this lesion characterized as simple fluid with a thin wall?
Q6	Is this lesion solid tissue demonstrating dark T2WI and dark DWI signals?
Q7	Does this lesion contain lipid content without restricted diffusion in its solid component?
Q8	What is the component type of this lesion?
Q9	What are the components of the lesion?
Q10	What are the patient's menopausal status and lesion size?
Q11	Are there peritoneal, mesenteric or omental nodularity or irregular thickening, with or without ascites?
Q12	Does the solid component of this lesion represent SA papillary structures?
Q13	Is the solid component of the lesion with restricted diffusion?
Q14	What is the type of solid components?
Q15	What is the ADC value of the cystic fluid?
Q16	Are there peritoneal, mesenteric or omental nodularity or irregular thickening, with or without ascites?
Q17	Is the solid component of the lesion with restricted diffusion?

Supplementary Table S5. Inter-observer agreement for standard O-RADS, modified non-contrast O-RADS and morphological features for differentiating benign and malignant adnexal masses

	Kappa value	95% CI	*P* value
O-RADS MRI score in the training set	0.859	0.811-0.908	<0.001
Modified non contrast O-RADS score in the training set	0.819	0.770-0.868	<0.001
Modified non contrast O-RADS score in the internal validation set	0.834	0.782-0.886	<0.001
Modified non contrast O-RADS score in the external validation set	0.811	0.753-0.868	<0.001
Peritoneal, mesenteric or omental nodularity or irregular thickening	0.723	0.358-1.088	<0.001
Lipid component	0.737	0.458-1.015	<0.001
Solid component morphological features	0.846	0.762-0.929	<0.001
Solid component limited diffusion	0.718	0.469-0.967	<0.001
Unilocular/multilocular	0.886	0.734-1.038	<0.001
Cystic fluid type	0.831	0.650-1.012	<0.001

O-RADS Ovarian-Adnexal Reporting and Data System, *95% CI* 95% Confidence interval
